# Reprogramming of synovial macrophage metabolism by synovial fibroblasts under inflammatory conditions

**DOI:** 10.1186/s12964-020-00678-8

**Published:** 2020-11-30

**Authors:** Noritaka Saeki, Yuuki Imai

**Affiliations:** 1 Division of Laboratory Animal Research, Advanced Research Support Center, Ehime University, Shitsukawa, Toon, Ehime 791-0295 Japan; 2 Division of Integrative Pathophysiology, Proteo-Science Center, Graduate School of Medicine, Ehime University, Shitsukawa, Toon, Ehime 791-0295 Japan; 3 Department of Pathophysiology, Graduate School of Medicine, Ehime University, Ehime, Japan

**Keywords:** Rheumatoid arthritis, Synovial macrophage, Synovial fibroblast, Metabolic reprogramming, Chronic inflammation

## Abstract

**Background:**

Macrophages adapt to microenvironments, and change metabolic status and functions to regulate inflammation and/or maintain homeostasis. In joint cavities, synovial macrophages (SM) and synovial fibroblasts (SF) maintain homeostasis. However, under inflammatory conditions such as rheumatoid arthritis (RA), crosstalk between SM and SF remains largely unclear.

**Methods:**

Immunofluorescent staining was performed to identify localization of SM and SF in synovium of collagen antibody induced arthritis (CAIA) model mice and normal mice. Murine arthritis tissue-derived SM (ADSM), arthritis tissue-derived SF (ADSF) and normal tissue-derived SF (NDSF) were isolated and the purity of isolated cells was examined by RT-qPCR and flow cytometry analysis. RNA-seq was conducted to reveal gene expression profile in ADSM, NDSF and ADSF. Cellular metabolic status and expression levels of metabolic genes and inflammatory genes were analyzed in ADSM treated with ADSM-conditioned medium (ADSM-CM), NDSF-CM and ADSF-CM.

**Results:**

SM and SF were dispersed in murine hyperplastic synovium. Isolations of ADSM, NDSF and ADSF to analyze the crosstalk were successful with high purity. From gene expression profiles by RNA-seq, we focused on secretory factors in ADSF-CM, which can affect metabolism and inflammatory activity of ADSM. ADSM exposed to ADSF-CM showed significantly upregulated glycolysis and mitochondrial respiration as well as glucose and glutamine uptake relative to ADSM exposed to ADSM-CM and NDSF-CM. Furthermore, mRNA expression levels of metabolic genes, such as *Slc2a1, Slc1a5, CD36*, *Pfkfb1, Pfkfb3* and *Irg1*, were significantly upregulated in ADSM treated with ADSF-CM. Inflammation marker genes, including *Nos2, Tnf, Il-1b* and *CD86*, and the anti-inflammatory marker gene, *Il-10*, were also substantially upregulated by ADSF-CM. On the other hand, NDSF-CM did not affect metabolism and gene expression in ADSM.

**Conclusions:**

These findings suggest that crosstalk between SM and SF under inflammatory conditions can induce metabolic reprogramming and extend SM viability that together can contribute to chronic inflammation in RA.

**Video Abstract**

## Background

Inflammation is a biological defense system to maintain homeostasis, but excessive inflammation such as that present in autoimmune diseases like rheumatoid arthritis (RA) induces onset and progression of pathological status. Among immune cells, macrophages are heterogenous and play multiple roles in several tissues to control inflammation and maintain homeostasis [1]. Macrophage heterogeneity has been shown to depend on microenvironmental stimuli in tissue niches [2–5]. Two types of activated macrophages, classically (M1) and alternatively (M2) activated, contribute to inflammation. M1 macrophages are induced experimentally by IFN-γ and lipopolysaccharide (LPS) and exhibit pro-inflammatory functions. Meanwhile, M2 macrophages, which are induced by IL-4, IL-10 or IL-13, associate with anti-inflammation, wound healing and tissue repair [6, 7]. These characteristics have also been shown to be involved in metabolic reprogramming of macrophages [7, 8]. In M1 macrophages, glycolysis is enhanced although tricarboxylic acid (TCA) cycle is dysregulated at least two key points [6, 9, 10]. These metabolic features of M1 macrophages contribute to production of inflammatory cytokines and nitric oxide (NO) [7, 11] that are related to the cytotoxic activities of M1 macrophages. M2 macrophages have increased rates of mitochondrial respiration and fatty acid uptake. However, the studies that demonstrated the relationships between metabolic status and M1/M2 macrophage plasticity have been revealed largely in vitro experiments conducted under static conditions [7, 12]. Recent studies suggested that the metabolic status and functional phenotypes of macrophages in vivo are more complex and are dependent on tissue type and disease conditions [3, 5, 7, 12, 13]. Thus, macrophage behavior can vary with tissue-specific microenvironments such as those found in the peritoneal cavity [3] and in tumors [13], but the relationship between tissue microenvironment and metabolic status in macrophages in vivo remains unclear.

Rheumatoid arthritis (RA) is a chronic inflammatory autoimmune disease. Persistent inflammation in synovial joints leads to synovium hyperplasia and progressive bone destruction [14]. The synovium lies on the inner surface of the joint capsule and has two main types of resident cells, synovial macrophages (SM) and synovial fibroblasts (SF). Under normal conditions the synovial membrane is thin, but in inflammatory joint diseases the membrane thickness increases due to proliferation of synovial cells and infiltration of immune cells from the blood into synovial tissue [14]. In RA synovium, cell–cell interactions between SM and SF are reported to accelerate disease progression [15–17]. Activated SM produce pro-inflammatory cytokines to affect surrounding cells in a paracrine and/or autocrine manner, whereas activated SF produce various kinds of inflammatory mediators, including chemokines and osteoclastogenic-factors [15–17]. Thus, crosstalk between SM and SF could affect the inflammatory status of the RA synovium, although the mechanisms underlying regulation of SM plasticity including how SM metabolic status might be altered by the synovial microenvironment in RA is largely unknown.

In this study, we modified the method to isolate SM and SF derived from synovial tissue of normal and arthritis model mice and used both primary synovial cells to demonstrate that metabolic reprogramming and immunological activation of SM can be induced by secretory stimulations from SF under inflammatory conditions.

## Methods

### Antibodies

The primary antibodies used included: rabbit monoclonal antibody against F4/80 (Cell Signaling Technology, Cat. No. 70076); rat monoclonal antibody against ER-TR7 (Abcam, Cat. No. ab51824); rat monoclonal antibody against Podoplanin (Pdpn; Wako, Cat. No. 015-24111); mouse monoclonal antibody against Nos2 (iNOS; R&D systems, Cat. No. MAB9502); rat monoclonal antibody against Nos2 (BioLegend, Cat. No. W16030C); and goat polyclonal antibody against Tnf (Santa Cruz, Cat. No. sc-1347). The secondary antibodies used included: Alexa Fluor488-conjugated goat anti-rabbit IgG, Alexa Fluor555-conjugated donkey anti-goat IgG, Alexa Fluor568-conjugated goat anti-mouse IgG and Alexa Fluor568-conjugated goat anti-rat IgG purchased from Molecular Probes. Rat monoclonal antibody against CSF1 (A16063L) and CSF2 (MP1-22E9) were purchased from BioLegend for use as neutralizing antibodies. Flow cytometry antibodies used included: PE-conjugated rat antibody against F4/80 (TONBO, BM8.1); PE-conjugated rat antibody against CD11b (TONBO, M1/70); PE-conjugated armenian hamster antibody against CD3e (TONBO, 145-2C11); PE-conjugated rat antibody against Ly6G (BioLegend, RB6-8C5); FITC-conjugated rat antibody against CD45 (BioLegend, 30-F11).

### Experimental arthritis model mice

Female C57BL/6 mice were housed in a specific-pathogen-free facility under climate-controlled conditions with a 12-h light/dark cycle, and were provided with water and standard diet (MF, Oriental Yeast, Japan) ad libitum. To induce collagen antibody induced arthritis (CAIA), 5 mg of anti-collagen 2 monoclonal antibody cocktail (Chondrex, USA, Redmond) was administered on day 0, followed by 50 μg of LPS on day 3; both agents were given intraperitoneally. After 10 days of induction, swollen tissue was used for histological analysis and primary culture.

### Histological analysis

Normal and CAIA mice were anesthetized and then euthanized with reflux flow of PBS. For paraffin embedding of tissues, knee joint samples were fixed overnight with 4% PFA-PBS followed by decalcification with 0.5 M EDTA. The samples were embedded in paraffin after dehydration and the paraffinized samples were cut with a microtome (RM2255, Leica Biosystems) into 6–7 μm thick sections that were then deparaffinized. For hematoxylin–eosin (H.E.) staining, deparaffinized sections were stained with Carazzi’s hematoxylin for 10 min, followed by staining with eosin Y for 10 min. For immunofluorescent staining, antigen retrieval was performed by incubation in citrate buffer (pH 7.0) for 60 min at 80–90℃ followed by blocking with 1% BSA-0.02% Triton X-PBS for 60 min. Sections were then incubated with primary antibodies diluted 1:200 (anti-F4/80) or 1:100 (anti-Tnf, anti-Nos2, anti-ER-TR7 and anti-Pdpn) overnight at 4 °C After washing, sections were incubated with 5 μg/mL secondary antibodies (Alexa Fluor488-conjugated goat anti-rabbit IgG, Alexa Fluor555-conjugated donkey anti-goat IgG, Alexa Fluor568-conjugated goat anti-mouse IgG and Alexa Fluor568-conjugated goat anti-rat IgG) for 1 h at room temperature. All sections were stained with 4′,6-diamidino-2-phenylindole (DAPI; 5 μg/mL) mixed with the secondary antibodies.

### Isolation of murine primary synovial cells

To obtain primary synovial cells from murine ankle tissue, isolation of synovial fibroblasts (SF) and tissue resident macrophages were performed as previously reported with slight modification [18, 19]. Briefly, normal and CAIA ankles were harvested by dislocation and treated with 1 mg/mL collagenase type IV (Sigma, USA) in Dulbecco’s Minimum Essential Medium GlutaMax (DMEM GlutaMax, Gibco, USA) supplemented with 10% fetal bovine serum (FBS, Gibco) and 1% antibiotic–antimycotic solution (anti-anti, Gibco) for 1–2 h before filtration with a 40 μm cell strainer (Falcon). To obtain normal tissue-derived SF (NDSF) and arthritis tissue-derived SF (ADSF), filtered cells were cultured on a culture dish (TPP, Switzerland) that was pre-coated with collagen (Type I-C, Nitta gelatin, Japan) in DMEM GlutaMax supplemented with 10% FBS and 1% anti–anti solution. After 1 h, non-adherent cells were transferred to another collagen-non-coated dish and adherent cells on collagen-coated dish were cultured in new medium for 4–5 days to expand the numbers with medium change every 2 days. To obtain arthritis tissue-derived synovial macrophages (ADSM), the transferred cells including ADSM, ADSF and other lymphocytes were co-cultured. After 24 h, non-adherent cells were removed and adherent cells on collagen-non-coated dish were cultured for 1–2 weeks to expand the numbers with medium change every 2 days. Then, fibroblastic cells were detached by treatment with 0.05% trypsin in Hanks' Balanced Salt Solution (HBSS) and removed by washing gently. Macrophage-like cells remaining on the dish were maintained for several days in DMEM GlutaMax supplemented with 10% FBS and 1% anti–anti solution before use for some experiments. To collect conditioned medium (CM), SF and SM were first cultured in DMEM GlutaMax without FBS for 3 days and the spent medium were centrifuged at 2000×*g* for 10 min. Some resulting supernatants were pooled and used as the CM. All cells were cultured at 37 °C in a humidified atmosphere of 5% CO_2._

### Real time RT-PCR

Total RNA was extracted with Isogen (Nippon gene, Japan) and RNeasy spin column kits (Qiagen, USA). First-strand cDNA was synthesized from total RNA using PrimeScript RT Master Mix (Takara Bio, Inc.) and subjected to real time RT-PCR using TB Green Premix Ex Taq II (Takara Bio, Inc.) with Thermal Cycler Dice (Takara Bio, Inc.) according to the manufacturer’s instructions. Gene expression levels were normalized relative to those of the housekeeping gene *Rplp0*. Primer sequences for each gene are listed in Additional file 1: Table S1.

### Flow cytometry

Primary cultured ADSM were treated with anti-F4/80-PE, anti-CD11b-PE, anti-Ly6G-PE, anti-CD3e-PE (0.2 μg /1 × 10^6^ cells) and anti-CD45-FITC (0.5 μg /1 × 10^6^ cells) for 20 min at room temperature. After washing, expressions of cells surface markers were evaluated using flow cytometer Gallios (Beckman Coulter, USA). Data were analyzed using software FlowJo (Treestar Inc., USA).

### RNA-seq analysis

High quality total RNA was obtained from primary cultured ADSF, NDSF and ADSM using RNeasy spin column kits and verified using an Agilent 2100 Bioanalyzer. RNA-seq analysis was performed as previously described [20]. RNA sequence libraries were prepared using an Illumina TruSeq Stranded mRNA LT Sample Prep kit for ADSM and ADSF, and NEBNext Ultra II Directional RNA Library Prep kit (New England Biolabs) for NDSF according to the manufacturer’s instructions. The libraries were subsequently validated for an average size of about 305–360 bp using a 2100 Bioanalyzer and an Agilent DNA1000 kit. Sequencing of paired-end reads (75 bp) was performed with a MiSeq Reagent kit V3 150 cycle on a MiSeq system (Illumina). Sequence data were mapped on the mouse genome (mm10) using TopHat [21] and analyzed using Cufflinks [22]. Hierarchical cluster analysis and principal-component analysis (PCA) were performed by MeV [23]. Gene Ontology analyses were performed using DAVID Bioinformatics Resources 6.8 [24].

### Cell counting assay

ADSM were pre-cultured for 16 h in serum-free medium before replacement with CM. After culturing in CM for 0–48 h, cells were fixed with 4% PFA-PBS and stained with 5 μg/mL DAPI. Fluorescent images were obtained with a 10 × Plan Fluor objective lens using an ImageXpress (Molecular Devices). The average number of nuclei in 9 fields per well was calculated using MetaXpress software (Molecular Devices).

### Cellular metabolism assay

MTT (3-(4,5-dimethyl-2-thiazolyl)-2,5-diphenyltetrazolium bromide) assay kits (Nacalai Tesque, Japan) were used according to the manufacturer’s instructions. Cell culture was performed using the same conditions as for the Cell Counting assay. After culturing in CM for 0–48 h, ADSM were incubated in medium with 0.5 mg/mL MTT for 2 h and lysed with 0.04 M HCl in isopropyl alcohol. Absorbance at 570 nm was measured using a FluxStation 3 (Molecular Devices). Metabolic flux was measured using a Seahorse XFp Flux Analyzer (Seahorse Bioscience) as described previously [25]. Prior to starting the assay, ADSM were seeded into an 8-well Seahorse culture plate (2 × 10^4^ cells per well) in serum-free medium and pre-cultured for 24 h before culture in CM for 16 h. For oxygen consumption rate (OCR) analysis, cells were cultured for 1 h in DMEM supplemented with 10 mM glucose, 1 mM pyruvate and 2 mM glutamine (Seahorse Bioscience, pH = 7.4 ± 0.1) and equilibrated at 37 °C in a CO_2_-free atmosphere. After three basal measurements, 1 μM oligomycin, 0.5 μM carbonyl cyanide 4-(trifluoromethoxy) phenylhydrazone (FCCP) and 0.5 μM antimycin A/rotenone were sequentially injected into the plate. For analysis of the extracellular acidification rate (ECAR), cells were cultured for 1 h in DMEM supplemented with 2 mM glutamine and equilibrated at 37 °C in a CO_2_-free atmosphere. After three basal measurements, 10 mM glucose and 1 μM oligomycin were sequentially injected as described for OCR analysis.

### Glucose and glutamine uptake assay

ADSM were pre-cultured for 16 h in DMEM (Wako, High glucose) without FBS before CM was added directly to a final concentration of 50% and the cells were cultured for another 24 h. The glucose and glutamine concentrations in the medium were measured using a Glucose Assay Kit-WST (DOJINDO, Japan) and Glutamine Assay Kit-WST (DOJINDO), respectively. Absorbance at 450 nm was measured using a FluxStation3 (Molecular Devices) according to the manufacturer’s instructions.

### ELISA

ADSM were cultured in CM for 24 h. The concentration of Tnf protein in the medium was measured using a Mouse Tnf ELISA kit (BioLegend). Absorbance at 450 nm was measured using a FluxStation3 (Molecular Devices) according to the manufacturer’s instructions.

### Immunocytochemical staining

Culture inserts (Ibidi, Germany) were placed in glass-bottom dishes and ADSM were seeded on the inserts. Cells were then cultured in CM for 1 day before fixation with 4% PFA-PBS. The culture inserts were removed and the cells were permeabilized for 10 min with 0.5% Triton X-PBS and blocked by treatment with 1%BSA-0.02% Triton X-PBS. Primary antibodies were added at 1:100 (Alexa Fluor594-conjugated anti-Nos2) and incubated overnight at 4 °C. After washing, the cells were incubated with 5 μg/mL DAPI for 30 min at room temperature.

### Statistical analysis

Two-tailed unpaired Student’s *t*-test with Microsoft Excel was used to analyze differences between two groups. ANOVA followed by post-hoc Tukey’s test with SPSS (IBM) was applied to compare multiple groups. For all graphs, data are represented as the mean ± standard deviation. Statistical significance was accepted when *P* < 0.05.

## Results

### Localization of synovial macrophages and fibroblasts in hyperplastic synovium of CAIA mice

**Fig. 1:**
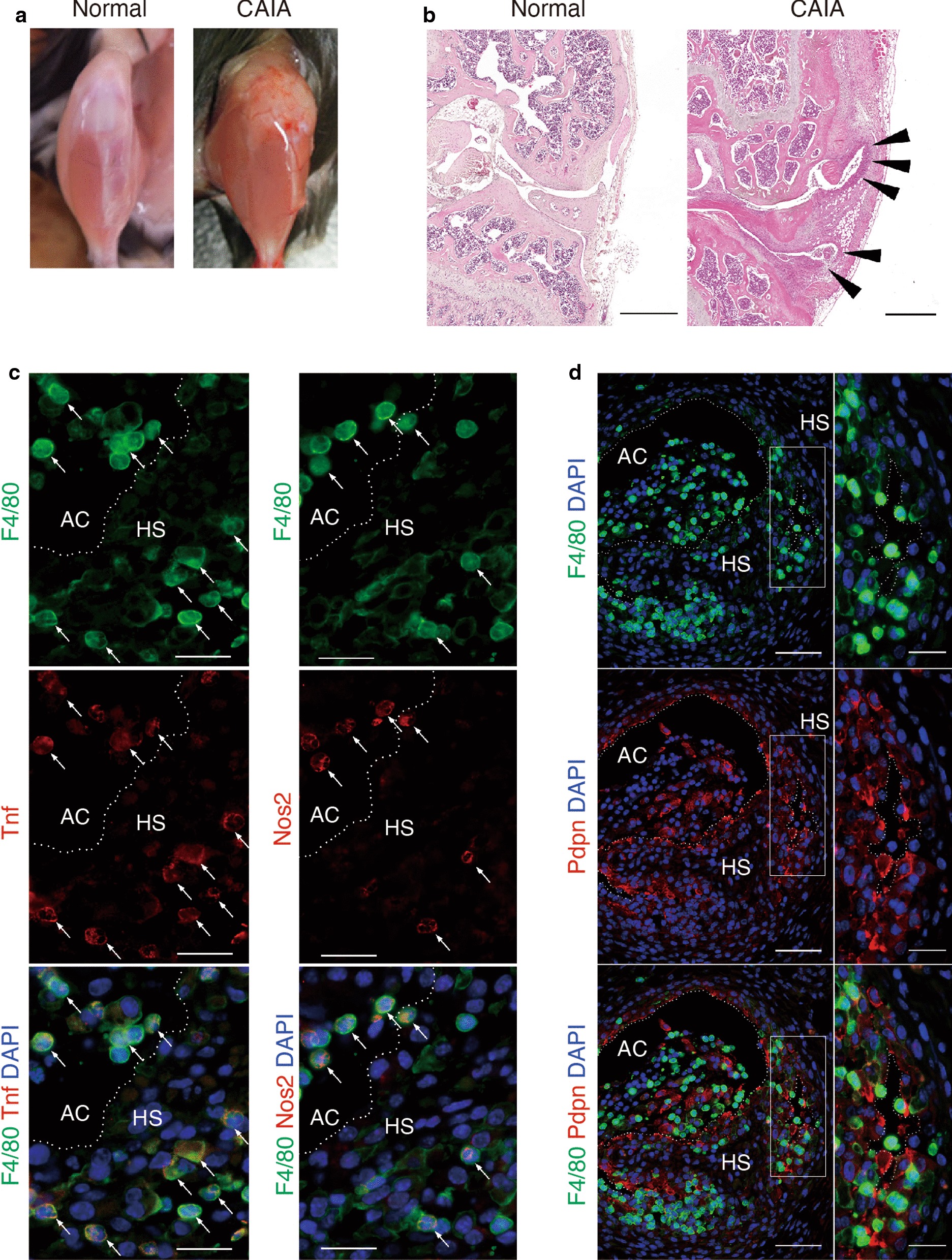
Histological analysis of knee joint tissue from CAIA mice. **a** Appearance of wild type mice with or without experimental arthritis (CAIA). **b** Sections of normal (left) and CAIA (right) knee joints stained with Hematoxylin and Eosin showing hyperplastic synovium (HS) in the joint (arrow head). Scale bar represents 500 μm. In immunofluorescent staining **c** F4/80-positive (green) cells were observed in the HS and articular cavity (AC). The inflammatory macrophage markers Nos2 and Tnf (red) co-localized in F4/80^high^-positive cells (arrow, yellow). Scale bar represents 20 μm. **d** Synovial cells were localized randomly in the HS. Panels on the right are high magnification images of boxed areas in the left panels. Podoplanin (Pdpn)-positive (red) cells were also observed in the HS. Scale bar represents 50 μm (left panel) and 20 μm (right panel). Histological data were technically replicated more than 2 times

The positional relationships between synovial macrophages (SM) and synovial fibroblasts (SF) have not been well characterized in mouse models of arthritis. To reveal localizations of both synovial cell types, we first established collagen antibody induced arthritis (CAIA) model mice and conducted histological analysis of joint tissues from these mice. The knee joints of CAIA mice exhibited remarkable swelling (Fig. 1a). Besides, H.E. staining of knee sections confirmed the presence of hyperplastic synovium that is characteristic of the arthritis phenotype (Fig. 1b). Immunofluorescent staining of the hyperplastic synovium from CAIA mouse knees revealed F4/80^high^- and F4/80^low^-positive SM were localized in regions adjacent to the inner surface of the synovium and articular cavity; in addition, inflammatory macrophage markers such as Tnf and Nos2 were expressed in F4/80^high^-positive SM (Fig. 1c). Cells that were positive for the inflammatory SF marker Podoplanin (Pdpn) were also located in a similar region (Fig. 1d). Synovial cells from CAIA mice were discontinuously and randomly distributed, but closely localized with each other in the inner region of the hyperplastic synovium while some SM were detached from SF (Fig. 1d). On the other hand, normal synovial membranes had few Tnf-positive SM and no detectable Nos2 (Additional file 2: Fig. S1A). Moreover, F4/80-positive SM continuously lined on ER-TR7-positive fibroblasts and no Pdpn was detected (Additional file 2: Fig. S1B). These data indicate that the normal spatial relationships between SM and SF were disrupted by arthritis and a de novo pathological microenvironment is associated with activation of SM in CAIA.

### Primary ADSF expresses genes associated with *secreted*

**Fig. 2:**
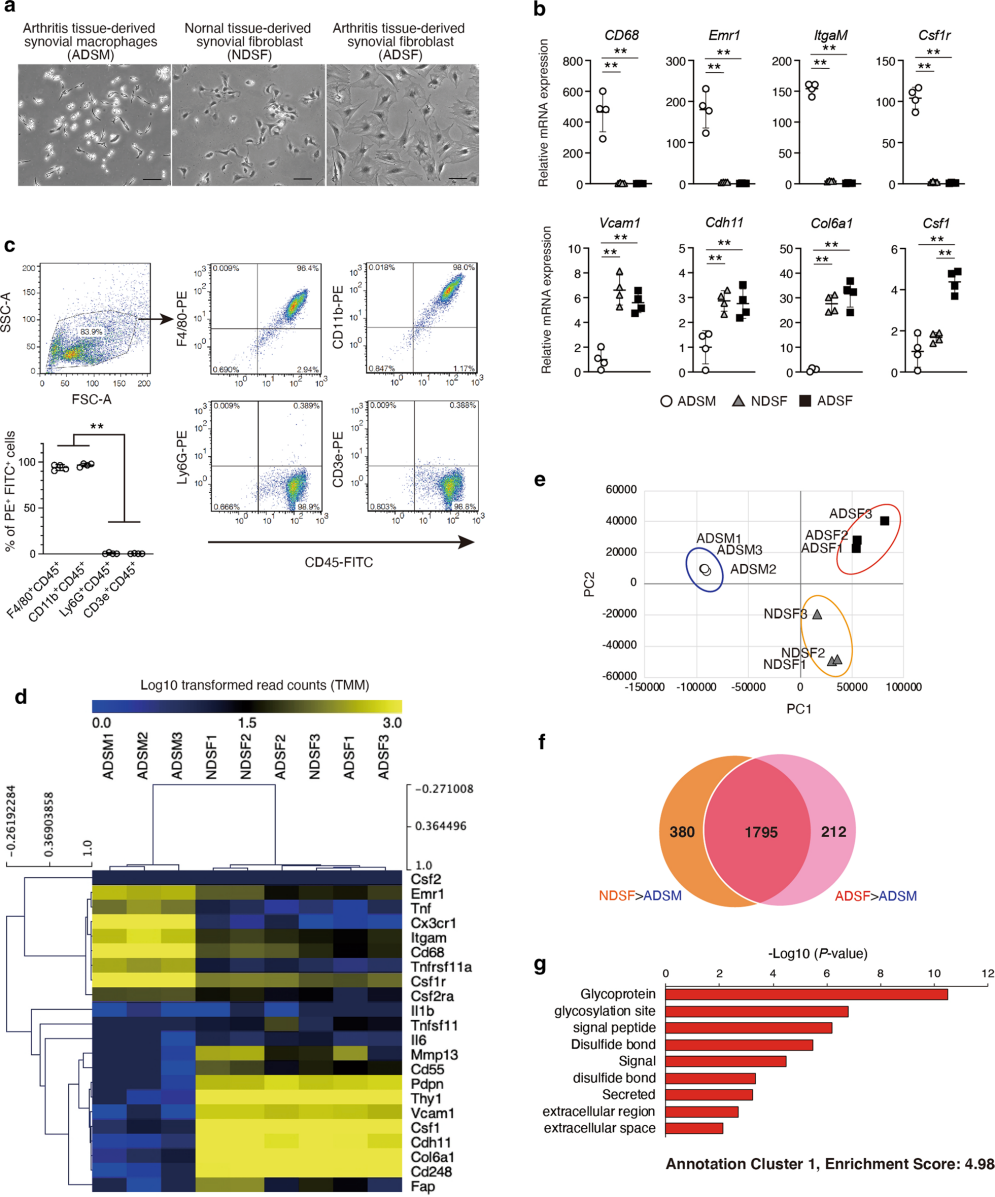
Gene expression profile in arthritis tissue-derived synovial macrophages (ADSM), normal tissue-derived synovial fibroblasts (NDSF) and arthritis tissue-derived synovial fibroblasts (ADSF). **a** ADSM, NDSF and ADSF were isolated from 4 independent ankles with or without CAIA. Representative phase-contrast images of ADSM, NDSF and ADSF. Scale bar represents 100 μm. **b** Gene expression of pan-macrophage and SF markers in ADSM, NDSF and ADSF were analyzed by RT-qPCR (*n* = 4). ** indicates *P* < 0.01 by ANOVA followed by Tukey’s test. Data are presented as average ± SD. **c** Pan-leukocyte marker (CD45), macrophage markers (F4/80, CD11b), B cell marker (Ly6G) and T cell marker (CD3e) were analyzed in ADSM by flow cytometry. Representative dot plots were shown. Percentage of FITC^+^ and PE^+^ were showed lower left graph (*n* = 4). ** indicates *P* < 0.01 versus Ly6G^+^ CD45^+^ and CD3e^+^ CD45^+^ by ANOVA followed by Tukey’s test. Data are presented as average ± SD. **d** Heatmap of selected genes in ADSM, NDSF and ADSF (*n* = 3). Log10 transformed read counts are scaled to 0.0 to 3.0. Rows and columns were ordered based on hierarchical clustering by MeV. **e** Principal-component analysis (PCA) displaying clusters of ADSM, NDSF and ADSF (*n* = 3). **f** Venn diagram for the number of specifically expressed genes in NDSF and ADSF. **g** Gene Ontology (GO) analyses were performed using DAVID Bioinformatics Resources. The top of enriched annotation cluster among 212 genes are illustrated by *P*-value. All data were obtained from 3–4 independent experiments using ADSM, NDSF and ADSF derived from 3 to 4 independent ankles with or without CAIA

The microenvironment in tissue niches affects cellular interactions to maintain or determine cell fate [2, 3, 5, 13, 26, 27]. To investigate cellular interactions between SM and SF, we generated primary cultures of bulk synovial cells from normal and CAIA ankle. To enrich the macrophage-like and fibroblast-like cells, isolation of SF and tissue resident macrophages were performed as previously reported with slight modification [18, 19]. We confirmed the enrichment of primary culture of macrophage-like cells from CAIA ankle and fibroblast-like cells from normal and CAIA ankle, even though we could not isolate enough amount of macrophage-like cells from normal ankle (Fig. 2a). Then, the expression of pan-macrophage and SF markers in respective synovial cells were analyzed by RT-qPCR (Fig. 2b). Pan-macrophage markers (*CD68, Emr1, ItgαM, Csf1r*) were expressed to significantly higher levels in macrophage-like cells than in fibroblast-like cells derived from normal and CAIA ankle. Meanwhile, the expression levels of SF markers (*Vcam1, Cdh11, Col6a1*) was significantly higher in both fibroblast-like cells compared to macrophage-like cells, and *Csf1* expression levels was significantly elevated in fibroblast-like cells derived from CAIA ankle compared to that derived from normal ankle (Fig. 2b). Flow cytometry analysis confirmed few other leukocytes were included in macrophage-like cells (Fig. 2c). These data verified the successful separation of arthritis tissue-derived SM (ADSM), normal tissue-derived SF (NDSF) and arthritis tissue-derived SF (ADSF). Next, we analyzed the gene expression profiles of ADSM, NDSF and ADSF by RNA-seq. Hierarchical cluster analysis of selected transcriptomes of pan-macrophage and SF markers revealed distinct gene expression patterns for ADSM and both SFs (Fig. 2d). In addition, principal-component analysis (PCA) also revealed separate gene clusters in ADSM, NDSF and ADSF (Fig. 2e). Recently, heterogeneous subsets of both SM and SF in synovium tissue of K/BxN serum transfer arthritis model mice has been reported using single-cell RNA-seq analysis [28, 29]. In these studies, SM and SF were classified 6 and 5 subsets, respectively. Thus, mRNA expression of major marker genes of each subset in isolated ADSM and ADSF were examined. RNA-seq data revealed that expression levels of *Fxyd2* (SM-subset 1 marker), *Cx3cr1* (SM-subset 4 marker), *Stmn1*, *Ube2c* and *Birc5* (SM-subset 5 marker) were relatively higher than other genes in ADSM (Additional file 2: Fig. S2A) and expression levels of *Sfrp2*, *Col11a1* (SF-subset 1 marker), *Inhba*, *Prg4* (SF-subset 2 marker), *Top2a*, *Hmgb2* and *Cdk1*(SF-subset 4 marker) were relatively higher than other genes in ADSF (Additional file 2: Fig. S2B). These data indicate that heterogeneity of isolated ADSM and ADSF may be lost and each cell population was changed into homogeneous with multiple subset properties under cultured condition. Comparing RNA-seq data sets after TMM normalization, the expression levels of 2175 genes in NDSF and 2007 genes in ADSF were more than 16-fold higher than in ADSM (value > MEDIAN and Log2 Fold-Change > 4). In addition, integrative analysis of the two data sets allowed extraction of 212 genes that were specifically expressed in ADSF (Fig. 2f). To characterize these genes, we performed Gene Ontology (GO) analyses using Database for DAVID bioinformatics resources. Top of functional annotation clustering was associated with secreted-related gene (Fig. 2g). Besides, GO biological process and KEGG pathway analysis revealed that immune response- and cytokine-related genes were enriched among ADSF specific genes (Additional file 2: Fig. S2C). These data suggest that secretory stimulation from ADSF is a key cellular interaction between SF and SM in the pathological synovial microenvironment.

### ADSF-CM induces glycolytic and oxidative metabolism in SM.

**Fig. 3:**
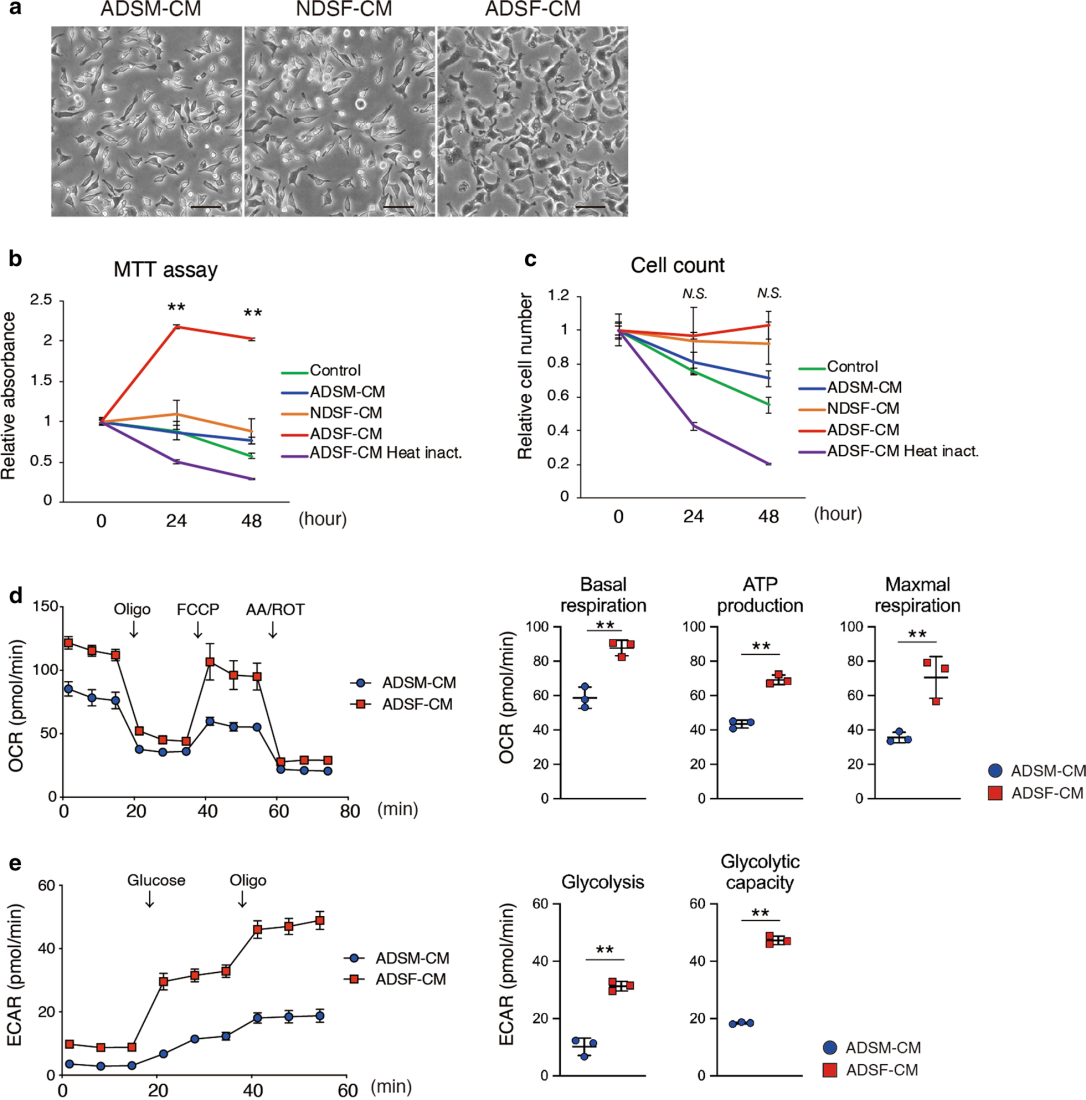
Cellular metabolism analysis in ADSM. **a** Representative phase-contrast images of ADSM cultured for 24 h by CM. Scale bar represents 100 μm. **b** Cellular metabolic status in ADSM was measured by MTT assay (*n* = 4). ** indicates *P* < 0.01 versus 0 h by ANOVA followed by Tukey’s test. **c** The number of nuclei were calculated to assess cell proliferation for 0–48 h (*n* = 4). *N.S.*; not significant versus 0 h by ANOVA followed by Tukey’s test. **d** Oxygen consumption rate (OCR) was assessed after the addition of oligomycin (Oligo), Carbonyl cyanide 4- (trifluoromethoxy) phenylhydrazone (FCCP) and antimycin A/rotenone (AA/ROT) at the indicated times (Left). Basal respiration, ATP production and maximal respiration were measured (Right) (*n* = 3). ** indicates *P* < 0.01 by unpaired *t*‐test. **e** Extracellular acidification rate (ECAR) was assessed after the addition of glucose and oligomycin (oligo) at the indicated times (Left). Glycolysis and glycolysis capacity were measured (Right) (*n* = 3). ** indicates *P* < 0.01 by unpaired *t*‐test. Data are presented as average ± SD. All data were technically replicated and repeated more than 2 times using ADSM derived from other CAIA ankle

We next investigated whether biological properties of ADSM were affected by factors secreted from ADSF. Morphological changes were observed when ADSM were cultured in serum-free conditioned medium from ADSF (ADSF-CM) (Fig. 3a). In addition, MTT assay revealed that formazan absorbance was significantly increased when ADSM was cultured in ADSF-CM for 24 and 48 h compared with that at 0 h (Fig. 3b). On the other hand, formazan absorbance was detected with constant levels in ADSM treated by NDSF-CM and slight time-dependent decreases when ADSM were treated with control medium, ADSM-CM, or heat-inactivated ADSF-CM within 48 h (Fig. 3b). Although formazan absorbance tended to correlate with cell number, nuclear staining revealed that the number of ADSM was not increased by incubation with ADSF-CM for 24 and 48 h (Fig. 3c). These results suggested that ADSF specific secreted factors could alter mitochondrial activity but not cell proliferation of ADSM. In addition, CSF1 and CSF2 are well-known cytokines that promote survival and proliferation of macrophage lineage cells [30, 31]. To investigate whether the effects of ADSF-CM are dependent on CSF1 and CSF2, we performed a neutralizing assay. Neutralizing antibodies against CSF1 and/or CSF2 failed to inhibit ADSF-CM-induced increases in formazan absorbance (Additional file 2: Fig. S3A), suggesting that novel secretory factors in ADSF-CM rather than CSFs can activate cell metabolic status and maintain ADSM viability. To examine metabolic alterations in ADSM associated with ADSF-CM treatment, we analyzed mitochondria stress and glycolysis profiles using an extracellular flux analyzer. There is no significant metabolic difference in ADSM treated with ADSM-CM or NDSF-CM, we examined metabolic influence of ADSF-CM with ADSM-CM as control (Additional file 2: Fig. S3B and S3C). Monitoring of oxygen consumption rate (OCR) in response to sequential additions of oligomycin (Oligo), carbonyl cyanide-4-(trifluoromethoxy)-phenylhydrazone (FCCP), and antimycin A plus rotenone (AA/ROT) showed significant increases in basal respiration, ATP production and maximum respiration rates in ADSM treated with ADSF-CM compared to treatment with ADSM-CM (Fig. 3d). Likewise, the extracellular acidification rate (ECAR) in response to sequential addition of glucose and oligo indicated that the glycolysis and glycolytic capacity were also significantly increased in ADSM treated with ADSF-CM relative to those treated with ADSM-CM (Fig. 3e). These data indicate that secretory factors from SF can alter the metabolic status of SM as characterized by enhanced glycolytic and oxidative metabolism that can activate and lengthen the lifespan of SM in the inflammatory synovial microenvironment.

### ADSF-CM induces metabolic reprogramming and inflammatory cytokine expression in ADSM

**Fig. 4:**
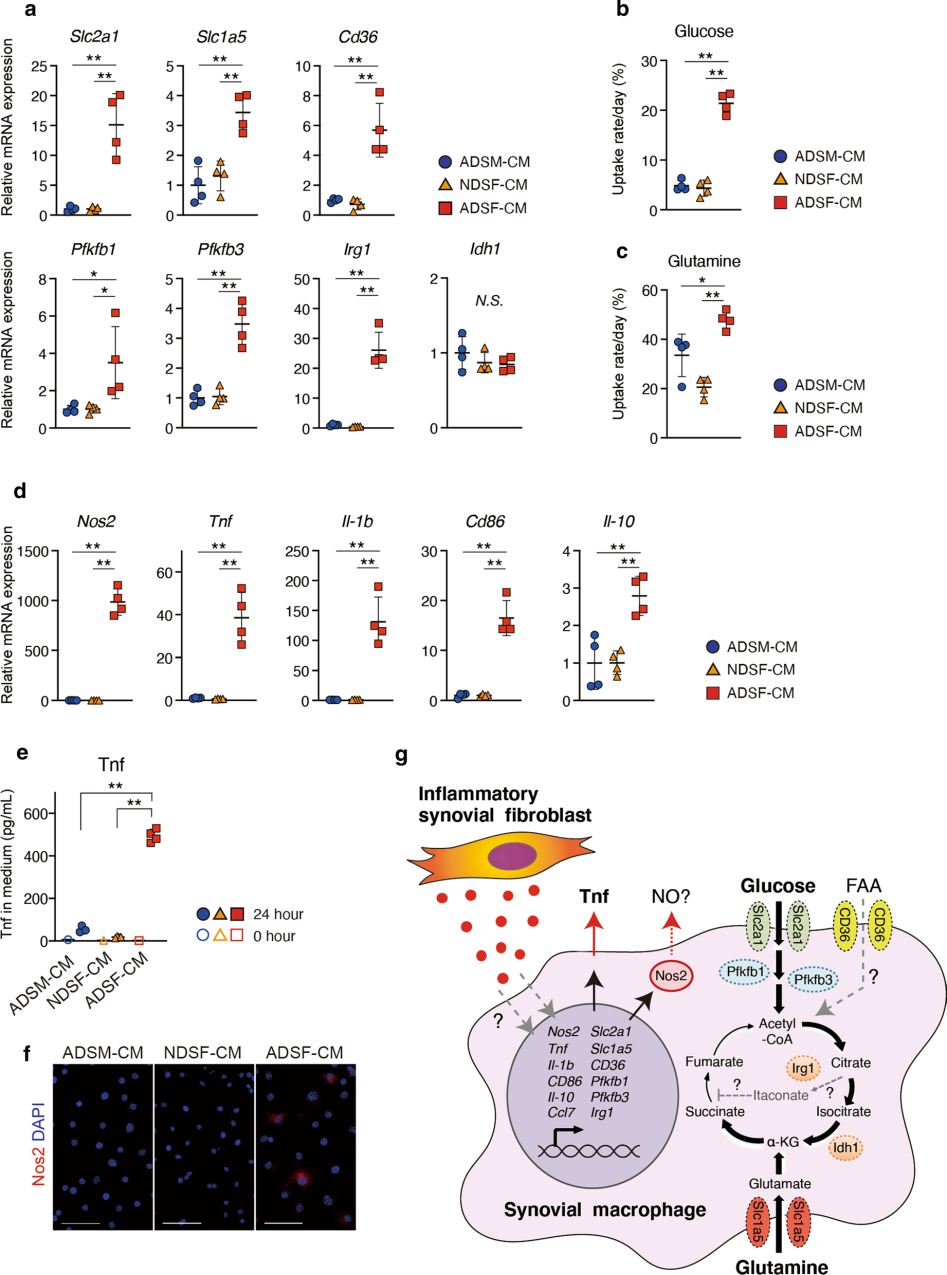
Metabolic reprogramming and immunological activation in ADSM. **a** Metabolic gene expression in ADSM treated with CM was analyzed by RT-qPCR (*n* = 4). **b** Glucose and **c** Glutamine uptake rate per 24 h were measured in ADSM treated with CM (*n* = 4). **d** Inflammatory and anti-inflammatory gene expression in ADSM treated with CM was analyzed by RT-qPCR (*n* = 4). **e** After culturing in CM for 0–24 h, the Tnf concentration in the medium was measured by ELISA (0 h; *n* = *1*, 24 h; *n* = 4). **f** Immunocytochemical staining of Nos2 (red) and DAPI (blue) in ADSM treated with CM. Scale bar represents 50 μm. **g** Summarized schema in this study. * and ** indicate *P* < 0.05 and *P* < 0.01, respectively, by ANOVA followed by Tukey’s test. Data are presented as average ± SD. All data were obtained from 4 independent experiments using ADSM derived from 4 independent CAIA ankles

To investigate how ADSF-CM facilitates metabolic reprogramming in ADSM, we performed RT-qPCR to examine mRNA expression levels of genes related to cellular metabolism in ADSM. The mRNA expression levels of transporter genes (*Slc2a1, Slc1a5, CD36*) and genes associated with glycolysis (*Pfkfb1, Pfkfb3)* were significantly upregulated in ADSM treated with ADSF-CM compared to ADSM treated with ADSM-CM and NDSF-CM (Fig. 4a). Among TCA cycle-related genes, mRNA expression levels of *Irg1* were also significantly upregulated but there was no difference in *Idh1* mRNA between ADSF-CM, NDSF-CM and ADSM-CM conditions (Fig. 4a). Uptake of glucose and glutamine by ADSM was significantly increased in the presence of ADSF-CM compared to ADSM-CM and NDSF-CM (Fig. 4b, c). These results indicate that ADSF-CM promotes mRNA expression of cellular metabolism related-genes and consequent metabolic reprogramming in ADSM that is required for immune response in macrophages [7, 8]. We next examined mRNA expression levels of several types of macrophage markers including inflammatory and anti-inflammatory genes in ADSM by RT-qPCR. Expression levels of major marker genes of heterogeneous SM-subsets were altered in ADSM treated with ADSF-CM as follows. *Aqp1*, *Fxyd2*, *H2-Ab*, *Cx3cr1*, *Stmn1* and *Acp5* were significantly downregulated in contrast with significant upregulation of *Ccl7* and *Ctsk* by ADSF-CM treatment compared to ADSM-CM and/or NDSF-CM treatment (Additional file 2: Fig. S4A). On the other hand, expression levels of *H2-Eb*, *Ube2c* and *Birc5* did not altered by ADSF-CM treatment (Additional file 2: Fig. S4A) and the expression levels of *Ccl8*, *Retnla*, *Sparc* and *Vsig4* were less than detection limit (data not shown). This result indicated that ADSM obtained the features of SM-subset 3, at least, by secretory factors from ADSF. In parallel, inflammatory macrophage markers such as *Nos2, Tnf, Il-1b* and *CD86* in ADSM were remarkably upregulated by ADSF-CM compared to ADSM-CM and NDSF-CM, whereas expression levels of the anti-inflammatory macrophage marker *Il-10* were slightly but significantly upregulated in ADSF-CM treated ADSM (Fig. 4d). On the other hand, there was no significant difference in expression levels of other inflammatory macrophage markers such as *Hif1a* and *Irf5,* or the anti-inflammatory macrophage markers such as *Pparg* and *Irf4* (Additional file 2: Fig. S4C). In addition, initial protein levels of Tnf in respective CM (0 h) were very low, but those in culture medium of ADSM treated with ADSF-CM for 24 h were significantly increased compared to that included in medium of cells treated with ADSM-CM and NDSF-CM. (Fig. 4e). Furthermore, immunocytochemical staining indicated that Nos2-positive cells were present only under ADSF-CM conditions (Fig. 4f). Taken together, secretory factors from SF alter gene expression to induce metabolic reprogramming in SM that subsequently can activate inflammatory responses in the arthritic synovial microenvironment (Fig. 4g).

## Discussion

RA is a major chronic inflammatory disease. The inhibitions of inflammatory response is effective therapeutic strategies for RA such as inhibitors of TNF, IL-6, CD86/80 or JAK as well as by disease-modifying anti-rheumatic drugs (DMARDs) [32]. Given the prominent role of inflammatory responses in RA, understanding the precise mechanisms underlying synovial inflammation, including the role of SM, which are main source of pro-inflammatory cytokine production, is critical. In RA synovium, pathological activation of synovial cells via cell–cell interactions can contribute to RA pathogenesis [15–17]. Both SM and SF have central roles in RA disease progression [15]. Therefore, in this study we investigated relationships between SM and SF and how these two cell types can contribute to pathogenesis of inflammatory arthritis such as RA using a CAIA murine arthritis model and primary cultures of synovial cells from these mice.

Here immunofluorescent staining revealed that the normal positional relationships between SM and SF, that orderly lined, was obviously disrupted in CAIA mice in a similar pattern with the previously reported K/BxN serum transfer arthritis (STA) model [28]. In the previous study, Culemann showed heterogeneity of SM in arthritic synovial tissue using single-cell RNA-seq and Cx3cr1^+^ lining macrophages form palisade-like structures to remove dying polymorphonuclear leucocytes in hyperplastic synovium surface of knee joint of K/BxN STA mice. However, such palisade-like structures were not observed at F4/80^+^ lining macrophages in that of CAIA mice. This finding indicates potential of difference between arthritis model types. As synovial cells were randomly localized in CAIA mice, some inflammatory SM (F4/80^high^, Nos2^+^, Tnf^+^) were distant from Pdpn^+^ SF in hyperplastic synovium. This result suggests that the synovial microenvironment in terms of SM and SF localization acquires a pathological structure in CAIA as well as K/BxN STA mice (Fig. 1). Furthermore, genome-wide gene expression profile analysis of primary cultures of murine synovial cells revealed that ADSF expresses abundant secreted related-genes including characterized genes as immune response, suggesting that interactions between SM and SF are mediated by secretory factors that play a key role to establish the pathological synovial microenvironment. Therefore, ADSM were cultured in ADSF-CM to mimic pathological synovial microenvironment instead of direct cell–cell interaction. Secretory stimulation induced by cytokines such as CSF1, CSF2 and IL-6 in SF is well established [15–17]. In particular, CSF1 and CSF2 are reported to be important molecules in the biological activities of macrophages, such as cell survival and cell differentiation; mesenchymal cells are the main sources of such cytokines [30, 31]. RT-qPCR analysis showed *Csf1* mRNA expression levels were higher in ADSF than in NDSF. However, here we found that neutralization of CSF1 and CSF2 did not inhibit metabolic activation of ADSM by ADSF-CM. In addition, *Il-6* expression was very low in cultured ADSF (data not shown). Furthermore, KEGG pathway analysis in SF specific gene set didn't enriched RA-related genes (mmu05323). Collectively, these data indicate that novel molecules, rather than CSF1, CSF2 and IL-6 from ADSF, likely affect metabolic activation in ADSM. Additional research will be needed to identify these novel secretory molecules from ADSF.

Metabolic reprogramming can affect signaling pathways and contribute to immunological response in macrophages [7, 11]. Changes in metabolic activity in M1 macrophages are manifested as enhanced glycolysis in response to upregulation of Slc2a1 (Glut1) and Pfkfb3 expression, and dysregulation of the TCA cycle associated with upregulation of Irg1 and downregulation of Idh1 [6, 33–35]. In M2 macrophages, expression of Pfkfb1, which has low phosphofructokinase activity, is upregulated and the uptake of glutamine and fatty acids is enhanced by upregulation of Slc1a5 (ASCT2) and CD36 respectively, resulting in upregulation of both glycolysis and mitochondrial respiration [36–38]. In the present study, metabolic flux analyses showed that both glycolysis and mitochondrial respiration are upregulated in ADSM cultured with ADSF-CM, suggesting that ADSM could have a long-lived phenotype. In addition, uptake of both glucose and glutamine was facilitated and expression levels of genes encoding pro-inflammatory macrophage markers such as *Tnf, Il-1b* and *Nos2* were simultaneously and significantly upregulated in ADSM cultured in ADSF-CM. These results indicate that interactions mediated by secretory factors from ADSF in the pathological synovial microenvironment could induce specific phenotypes in SM, which have both long-lived and pro-inflammatory features to induce chronic inflammation in RA. On the other hand, it has been reported that SM in RA express HIF1A that is important transcription factor to promote macrophage activation [7, 39, 40], however, *Hif1a* mRNA was not significantly upregulated in ADSM treated with ADSF-CM in this study. As previous reports have reported that citrate and succinate accumulation can stabilize Hif1a [7], we should analyze protein stability of Hif1a in the future.

Recently, macrophage niches in various tissues have been reported [2, 3, 5, 13, 26]. Therefore, the pathological synovial microenvironment populated by SM and SF could also be termed an inflammatory synovial niche. Given that metabolic reprogramming contributes to immunological responses through such as Hif1a stability and epigenetic alterations in immune cells including macrophages [7, 8, 41–43], metabolic realignment in immune cells has received attention as a therapeutic target [44, 45]. However, we could not reveal causal link between the changes of metabolic activity and of inflammatory gene expression in ADSM. Therefore, future studies are needed to elucidate relationships between metabolism alterations, stabilization of transcription factor such as Hif1a and epigenetics alterations in SM to discover novel therapeutic strategies for RA that involve targeting of SM metabolism.

Metabolic feature of resident macrophages has been studied in some microenvironment conditions [3, 13], however, that of SM in arthritis models and RA patients were not well studied. This might be caused by the difficulty of isolation of SM with high-purity. There are some reports for metabolic status in RA macrophage, which are generally derived from peripheral monocytes [46, 47]. In the present study, we established simple method to isolate not only murine SF but also SM derived from swollen ankle of arthritis model mice, although we could not isolate enough amount of SM derived from normal ankle. This method would be useful for in vitro experiments of SM in inflammatory arthritis at least mice models. Overall, this study does have some limitations. First, the results are limited to in vitro experiments and the ADSM phenotypic alterations associated with ADSF-CM were conducted under serum-free conditions to eliminate the effects of residual FBS in CM. Therefore, additional investigations are needed to assess the ADSM phenotype under different culture conditions to reveal the pathological niche in greater detail. Second, isolated ADSM and ADSF might seem to lost their heterogeneity of cell population by influence of in vitro culture condition because expression patterns of marker genes in isolated ADSM and ADSF could not be categorized into any subsets, which were previously reported [28, 29]. Meanwhile, slight possibility was shown that ADSF-CM treatment induced polarization into SM-subset 3-like cells. More experiments are needed to clarify which SF-subset can secrete the factors to induce metabolic reprogramming in SM and contribute differentiation to each subset of SM.

## Conclusions

In conclusion, this study explored the secretory interactions between SM and SF in the pathological synovial niche of inflammatory arthritis that can enhance SM activation and increase the lifespan of these cells via metabolic reprograming under inflammatory conditions.

## Supplementary information

**Additional file 1.** Supplemental Table of primer sequence for RT-qPCR.

**Additional file 2.** Supplemental figure 1–4.

## Data Availability

Data sets from RNA-seq were deposited in the NCBI Gene Expression Omnibus under accession numbers GSE142607.

## Abbreviations

AA/ROT: Antimycin A plus rotenone
ADSF: Arthritis tissue-derived synovial fibroblasts
ADSM: Arthritis tissue-derived synovial macrophages
BSA: Bovine serum albumin
CAIA: Collagen antibody induced arthritis
CM: Conditioned medium
CSF: Colony stimulating factor
DAPI: 4′,6-Diamidino-2-phenylindole
DMARD: Disease-modifying anti-rheumatic drug
DMEM: Dulbecco’s minimum essential medium
ECAR: Extracellular acidification rate
ELISA: Enzyme-linked immunosorbent assay
FBS: Fetal bovine serum
FCCP: Carbonyl cyanide-4-(trifluoromethoxy)-phenylhydrazone
FPKM: Fragments per kilobase of exon per million reads mapped
GO: Gene ontology
H.E.: Hematoxylin–eosin
HBSS: Hanks' balanced salt solution
LPS: Lipopolysaccharide
MTT: 3-(4,5-Dimethyl-2-thiazolyl)-2,5-diphenyltetrazolium bromide
NDSF: Normal tissue-derived synovial fibroblasts
NO: Nitric oxide
OCR: Oxygen consumption rate
Oligo: Oligomycin
PCA: Principal-component analysis
Pdpn: Podoplanin
RA: Rheumatoid arthritis
RT-qPCR: Real-time quantitative polymerase chain reaction
SD: Standard deviation
SF: Synovial fibroblasts
SM: Synovial macrophages
STA: Serum transfer arthritis
TCA: Tricarboxylic acid
TMM: Trimmed mean of M values
Tnf: Tumor necrosis factor
