# Supplementary material for: Reprogramming of synovial macrophage metabolism by synovial fibroblasts under inflammatory conditions
Source: Cell Commun Signal. 2020 Nov 30;18:188. doi: 10.1186/s12964-020-00678-8 (PMC7708128; doi:10.1186/s12964-020-00678-8)
Supplement: Supplementary file 3 — Additional file 2. Supplemental figure 1–4. [file 12964_2020_678_MOESM3_ESM.pdf]

A

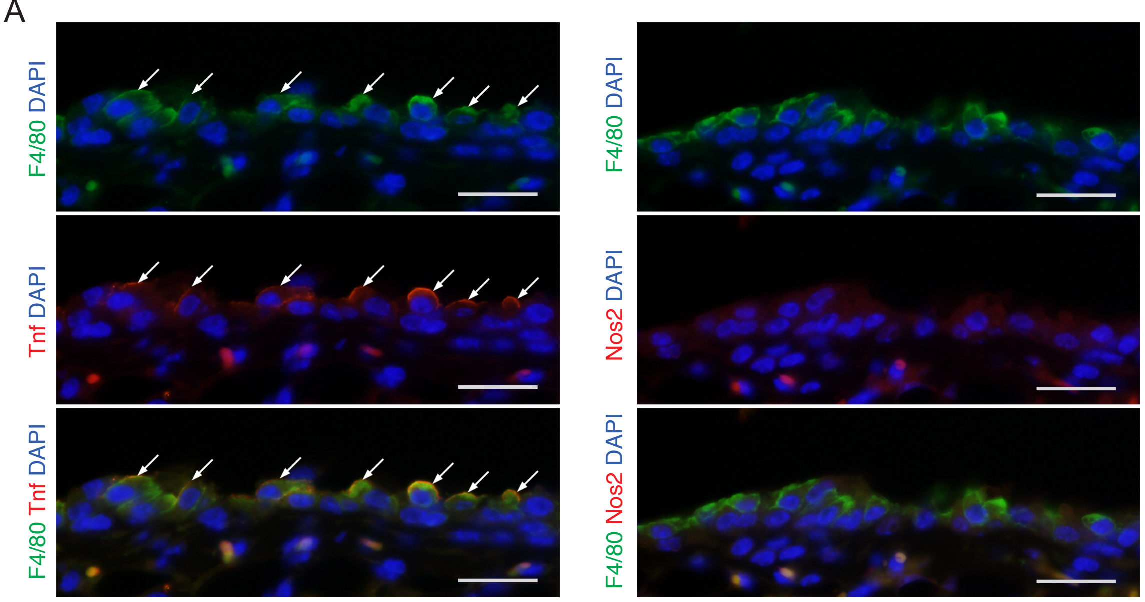

B

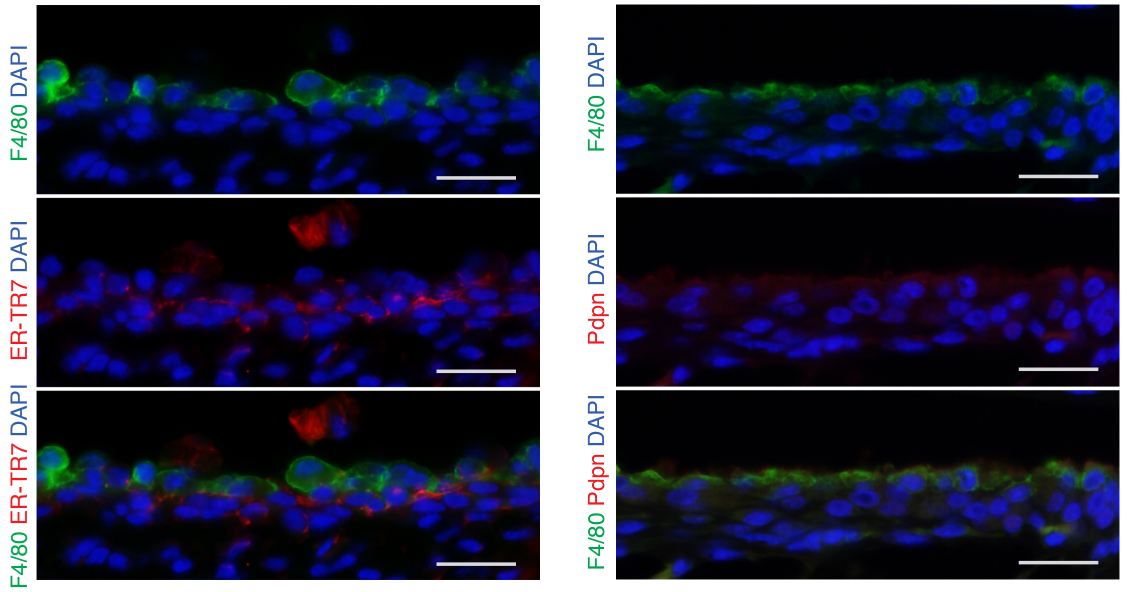

Supplemental Figure S1
Histological analysis of normal knee joint tissue. (A) Tnf (red) signals were detected on some F4/80-positive (green) cells (arrow). Nos2 was not detected. Scale bar represents 20 μm. (B) F4/80-positive (green) macrophages lined on ER-TR7-positive (red) fibroblasts in the synovium. Among inflammatory synovial fibroblast markers, Podoplanin (Pdpn) was not detected. Scale bar represents 20 μm. Histological data were technically replicated more than 2 times.

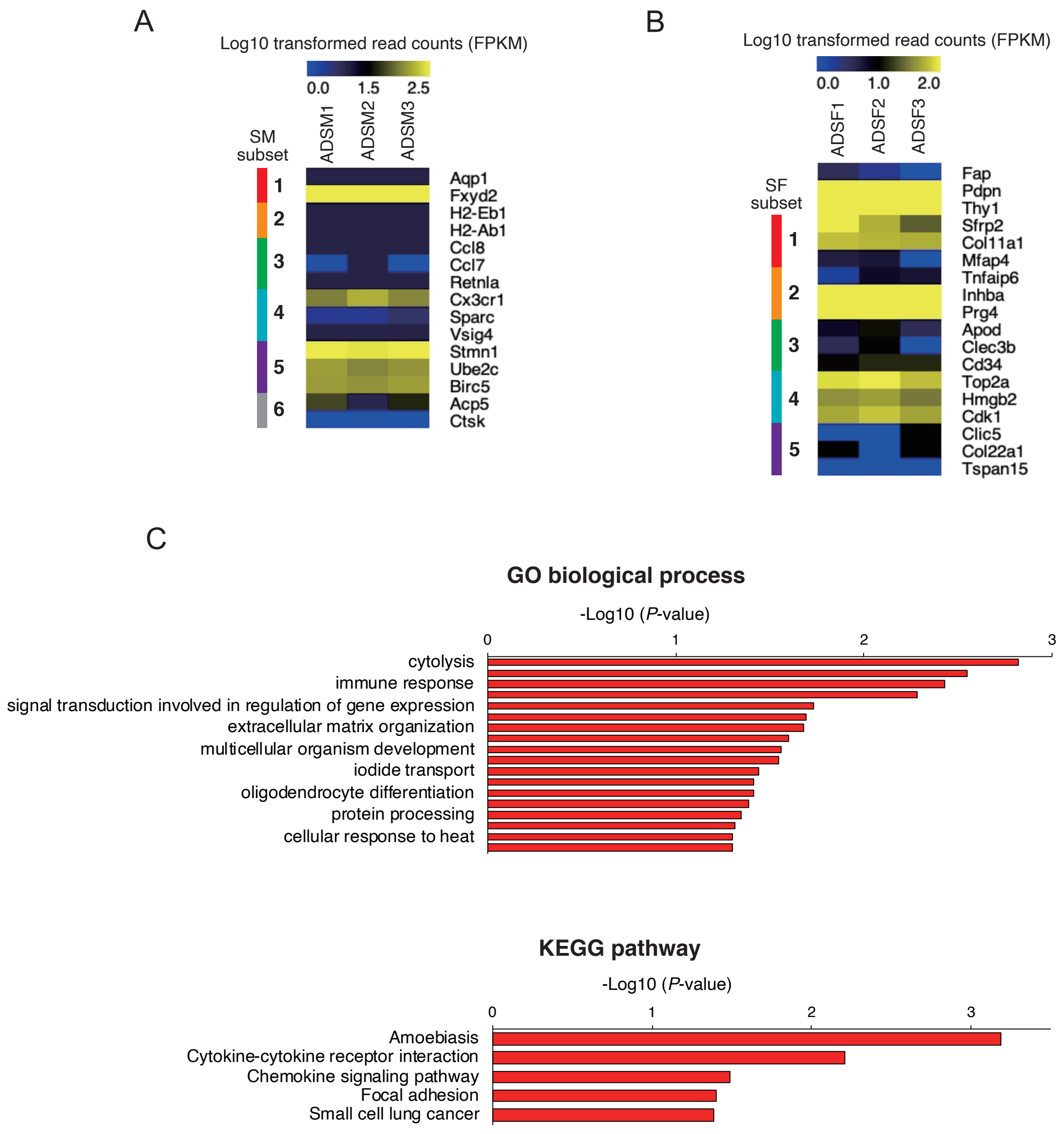

Supplemental Figure S2

RNA-seq data analysis (A) Heatmap of SM-subset marker genes in isolated ADSM. Log10 transformed read counts are scaled to 0.0 to 2.5. (*n*=3) (B) Heatmap of SF-subset marker genes in isolated ADSF. Log10 transformed read counts are scaled to 0.0 to 2.0. (*n*=3). (C) Gene Ontology (GO) analyses were performed using DAVID Bioinformatics Resources. GO biological process (upper) and KEGG pathway (lower) are displayed by *P*-value. All data were obtained from 3 independent experiments using ADSM, NDSF and ADSF derived from 3 independent ankles with or without CAIA.

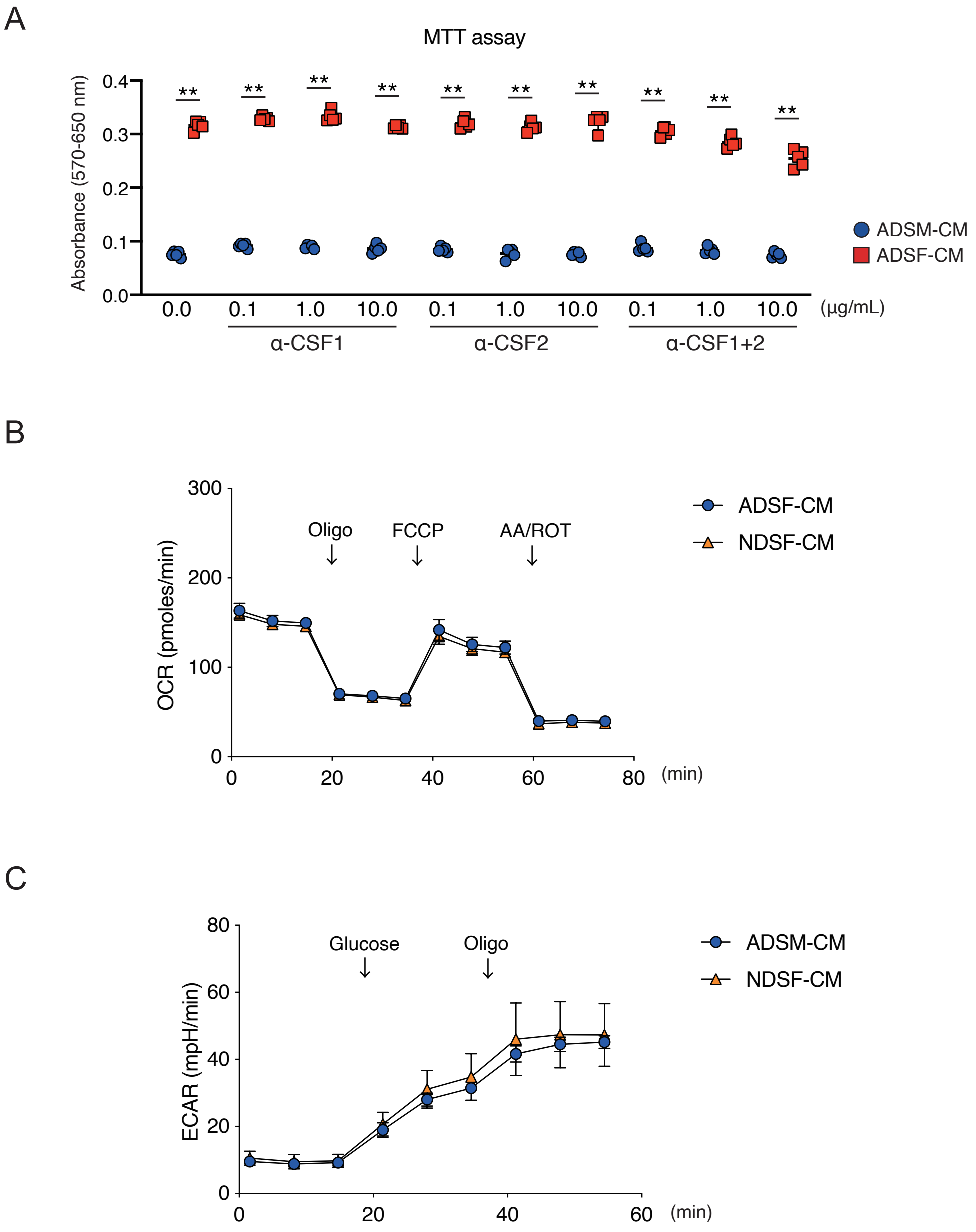

Supplemental Figure S3

Cellular metabolism analysis in ADSM. (A) neutralizing antibody against CSF1 and CSF2 (A16063L and/or MP1-22E9) was added to the ADSM-CM and ADSF-CM. After 24 hours, MTT assay was performed (*n*=5). ** indicates *P*<0.01 against ADSM-CM by unpaired t - test. Data are presented as average ± SD.(B) Oxygen consumption rate (OCR) was assessed after the addition of oligomycin (Oligo), Carbonyl cyanide 4- (trifluoromethoxy) phenylhydrazone (FCCP) and antimycin A/rotenone (AA/ROT) at the indicated times in ADSM treated with NDSF-CM (*n*=3). (C) Extracellular acidification rate (ECAR) was assessed after the addition of glucose and oligomycin (oligo) at the indicated times in ADSM treated by NDSF-CM (*n*=3). Data were technically replicated and repeated more than 2 times using ADSM derived from other CAIA ankle.

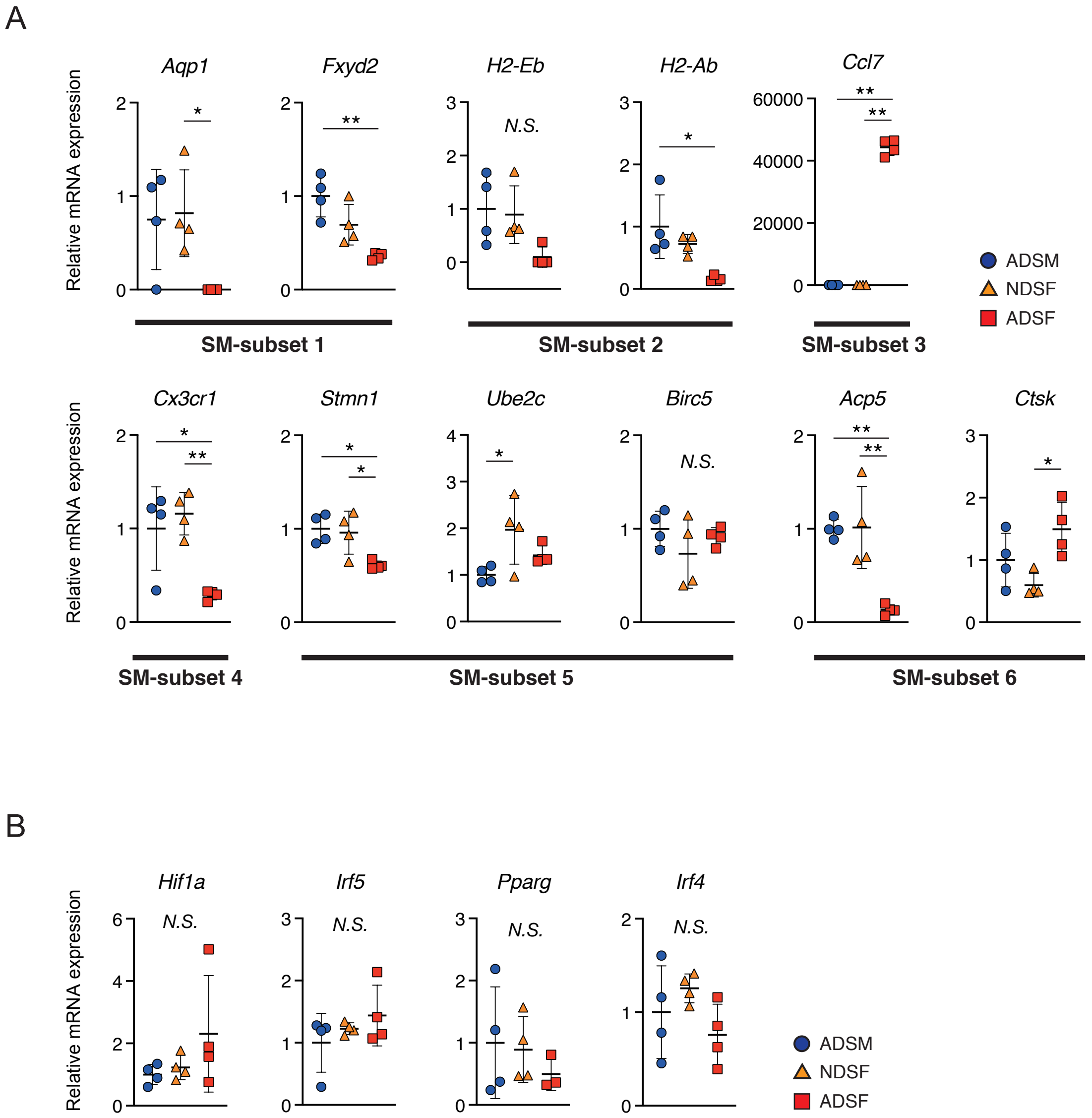

Supplemental Figure S4

Gene expression in ADSM treated with CM. (A) SM-subset marker gene in isolated ADSM treated with CM was analyzed by RT-qPCR (*n*=4). (B) Inflammatory and anti-inflammatory gene expression in ADSM treated with CM was analyzed by RT-qPCR (*n*=4). * and ** indicate *P*<0.05 and *P*<0.01, respectively, by ANOVA followed by Tukey’ s test. *N.S.*; not significant. Data are presented as average ± SD. All data were obtained from 4 independent experiments using ADSM derived from 4 independent CAIA ankles.
